# Prognostic Factors in 26 Cats Undergoing Surgery for Extra-Hepatic Biliary Obstruction

**DOI:** 10.3390/vetsci11120610

**Published:** 2024-11-29

**Authors:** Jonathan P. Speelman, Ki-Lam Hui, Nicolas T. Woodbridge, Susanne Pfeiffer, Julia A. Beatty, Alan H. Taylor

**Affiliations:** 1 Advanced Vetcare, 2 Chandler Hwy, Kew, Melbourne, VIC 3101, Australia; 2 Pulse Veterinary Specialists and Emergency, 450 Ordze Road, Sherwood Park, AB T8B 0C5, Canada; karenh@pulseveterinary.ca; 3 Dick White Referrals, Station Farm, London Road, Six Mile Bottom, Cambridgeshire CB8 0UH, UK; nwoodbridge1@gmail.com; 4 Jockey Club College of Veterinary Medicine and Life Sciences, City University of Hong Kong, Hong Kong SAR, China; 5 Centre for Animal Health and Welfare, City University of Hong Kong, Hong Kong SAR, China; 6 Department of Veterinary Clinical Sciences, Jockey Club College of Veterinary Medicine and Life Sciences, City University of Hong Kong, Hong Kong SAR, China; 7 CityU VMC, Sham Shui Po, Kowloon, Hong Kong SAR, China; alantaylor@cityuvmc.com.hk

**Keywords:** feline, biliary obstruction, hyperbilirubinemia, hypotension, biliary surgery, cholelithiasis, cholecystectomy

## Abstract

Surgical management of feline extra-hepatic biliary obstruction (EHBO) has poor survival rates with few prognostic factors reported in the literature. The etiology and clinical findings of feline EHBO and their influence on short-(2 weeks–6 months) and long-term (>6 months) survival and prognosis were examined in an observational clinical retrospective study of 26 client-owned cats undergoing surgery for biliary obstruction at one institution between 2012 and 2020. The etiology of EHBO was determined in 21/26 cats, which included inflammatory causes (14/21), neoplastic causes (6/21), and a duodenal foreign body (1/21). Pre-operative hyperbilirubinemia and post-extubation hypotension (mean arterial pressure < 60 mmHg) were statistically associated with reduced short- and long-term survival. Short-term survival was documented in 17/26 cats, and long-term survival in 13/26 cats. Median survival time (MST) was 86 days (range, 0–1497). An MST of 17 days (range, 2–520) in cats with malignancies was found compared to an MST of 1165 days (range, 61–2268) in EHBO resulting from inflammatory complexes. Pre-operative hyperbilirubinemia and immediate post-operative hypotension may represent prognostic factors for cats undergoing surgery for EHBO. Cats with inflammatory causes of EHBO carry a more favorable prognosis than neoplastic causes. Further studies are required to evaluate the validity of the observed associations.

## Simple Summary

Cats with obstruction of bile flow from the gall bladder to the intestine have traditionally had poor survival rates following surgery. Clinical signs associated with the condition and different causes were examined in 26 client-owned cats. Cats with high levels of bilirubin in the blood before surgery or those with low blood pressure after surgery had poor short- and long-term survival. In total, 17 of the 26 cats survived in the short term, and 13/26 survived in the long term. Cats with cancer as the cause of obstruction had a median survival time of 86 days (range, 0–1497), and cats with other causes had a median survival time of 1165 days (range, 61–2268). Some cats had normal blood results despite their biliary obstruction. Further studies involving larger numbers of cats would be needed to confirm if high levels of bilirubin or low blood pressure are indeed indicators for low survival rates in cats undergoing surgery for this condition.

## 1. Introduction

Extra-hepatic biliary obstruction (EHBO) is an uncommon and potentially life-threatening condition in cats that is associated with conditions that obstruct bile flow from the liver and gallbladder to the duodenum [1,2,3,4,5]. Reported common causes of EHBO in cats include inflammatory and stone-forming conditions of the gall bladder or common bile duct (CBD), or neoplastic processes involving the CBD, liver, pancreas or duodenum [6,7,8,9,10,11,12]. Less common causes of EHBO in cats include proximal duodenal foreign bodies [13], a grass awn [8], hair [14], diaphragmatic herniation [15], parasite infestation [16,17], and bilirubin cholelithiasis [18]. Gallbladder mucocele is a common cause of EHBO in dogs [19] but is rarely reported in cats [20,21,22]. Fewer mucus-producing glands in the feline compared with the canine gallbladder may explain this apparent species difference [1,23].

Reported consequences of EHBO include hypotension, acute kidney injury, myocardial impairment, gastrointestinal ulceration and hemorrhage, coagulopathy, and delayed wound healing [6,7,24,25,26,27,28,29,30]. The prognosis of cats undergoing extra-hepatic biliary surgery has been historically viewed as guarded, with short-term mortality rates ranging from 36% to 50% [6,7,31]. Simpson et al. and Mayhew et al. reported an improved prognosis when cholecystectomy was included in the surgical management of feline patients with EHBO of non-neoplastic causes compared to those who did not have a cholecystectomy [6,12]. Boute et al. also demonstrated a longer median survival time in surgically managed feline EHBO patients associated with chronic inflammatory disease compared to neoplastic diseases of the hepatobiliary system, though no statistical significance was found [7]. Griffin et al. suggested that temporary choledochal stenting was an effective treatment modality in cats with EHBO with few procedural complications and potential for prolonged survival, though substantial risk for recurrence of EHBO after stents had passed into the gastrointestinal tract was identified [4]. No other prognostic factors have been identified in cats undergoing extra-hepatic biliary surgery.

The objective of this study was to describe the primary etiology and clinicopathological findings of extra-hepatic biliary obstruction (EHBO) confirmed during exploratory celiotomy. We also aimed to identify potential prognostic factors in cats undergoing surgery for EHBO through an exploratory analysis of numerous clinical variables and outcomes.

## 2. Method and Materials

### 2.1. Data Extraction and Collection

A retrospective review of medical records at the CityU Veterinary Medical Centre between March 2012 and July 2020 was carried out to identify cases of EHBO in cats. Keyword searches, including the terms extra-hepatic biliary obstruction, EHBO, cholestasis, hyperbilirubinemia, icterus and jaundice, cat, and feline, were performed in the digital medical software system (RxWorks^®^) (RxWorks^®^ veterinary software, Covetrus Global Software Services, https://software.covetrus.com/apac/veterinary-solutions/rxworks-veterinary-inventory-management/, accessed on 23 September 2024) to identify inclusions. Inclusion was based on simultaneous findings of hyperbilirubinemia and biliary dilatation with a common bile duct lumen size > 5 mm in diameter evident on abdominal ultrasound. This criterion was based on previous studies which found that a common bile duct diameter over 5 mm was present in 97% of the cats with extra-hepatic biliary obstruction [22]. EHBO was confirmed at surgery by a boarded surgeon as the observed dilatation of the CBD proximal to the duodenum and an inexpressible gallbladder. Cats were excluded if there was no dilatation of the CBD, no obvious extra-hepatic biliary obstruction, if they were operated by a non-boarded surgeon, or were managed conservatively.

### 2.2. Pre-Operative Clinical Findings

Data recorded included signalment, clinical signs at presentation, duration of clinical signs before surgical intervention, presence of concurrent medical conditions, diagnostic imaging findings, peri-operative medical treatments, and clinicopathologic test results. Deviation of blood parameters from normal was based on reference ranges provided by in-house Idexx^®^ blood testing machines (Idexx^®^ Blood test machines, included ProCyte Dx Hematology Analyzer, Catalyst One Chemistry Analyzer and VetStat Electrolyte Blood Gas Analyzer).

### 2.3. Anesthesia Records

Anesthesia records, including peri-operative blood pressures, were reviewed if available. Hypotension was defined as the mean arterial blood pressure being less than 60 mm Hg. All therapies used for the management of hypotension were recorded when available.

### 2.4. Intra-Operative Findings and Sampling

Intra-operative findings, surgical treatments, bacterial culture and sensitivity testing of bile sampled at surgery and histopathological findings from tissue samples obtained at surgery were recorded where available for all cats. Histopathological analysis was performed by board-certified pathologists from five different diagnostic laboratories.

### 2.5. Post-Operative Management

Post-operative monitoring, blood testing, medical treatments, complications, hospital stay length, survival time and cause of death, if applicable, were documented. Surgical complications were defined as any adverse event temporally associated with and attributed to surgical intervention [32]. The caregivers were contacted for consent to have their cat’s data included in this study, and follow-up information was collected if necessary.

### 2.6. Outcomes

For statistical purposes, non-survivors were defined as those cats dying within the first two weeks, and post-operative survivors were defined as either short-term (≥two weeks but <six months) or long-term (≥six months) survivors.

### 2.7. Determination of Etiology of EHBO

The etiology of EHBO was determined based on the clinical data available, and then categorized as i. inflammatory disease (i.e., pancreatitis, duodenitis, cholecystitis, cholangiohepatitis and/or other inflammatory disorders, including cholelithiasis); ii. benign neoplasia; iii. malignant neoplasia; iv. other (i.e., duodenal foreign body); or v. undetermined.

### 2.8. Statistical Analysis

**Figure 1:**
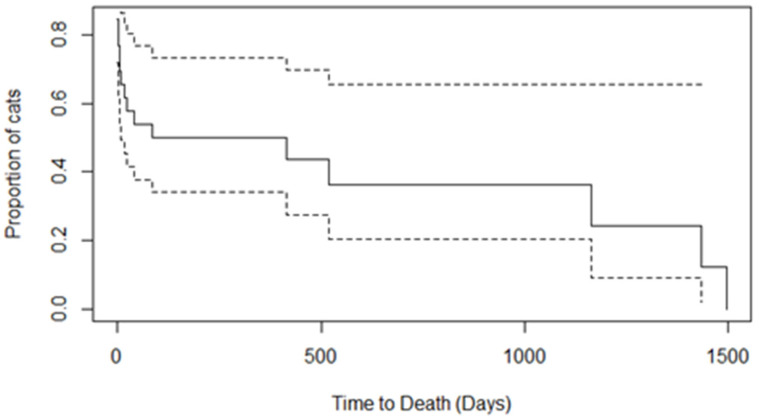
Kaplan–Meier survival curve for the survivorship function for the time from surgery to death. Note: Dashed lines represent the 95% confidence intervals. The median survival time for cats was 86 days post-surgery.

Statistical analysis was performed using computer software (IBM SPSS Statistics for Windows, Version 29.0. Armonk, NY, USA: IBM Corp). Descriptive statistics (median, minimum and maximum values) were derived for pre- and post-operative blood parameters, and median survival times were calculated. The continuous variables representing pre- and post-operative blood parameters, clotting profile, intra- and post-operative hypotension, inflammation and malignancy were categorized into two categories each and Fisher’s exact test was used to assess the association between these variables and short- and long-term survival. This was an exploratory analysis to find any associations that might require further investigation in future studies; so, no mathematical correction of the *p*-value was made for multiple comparisons [33]. Odds ratios were calculated to quantify the strength of the associations, and 95% confidence intervals were calculated using the Woolf method with the Haldane–Anscombe method for zero-cell correction (adding 0.5 to all cells in contingency tables containing at least one zero cell). Multivariate models were not applied due to unmet requirements relating to the sample size. A Kaplan–Meier curve was used to plot the survival time post-surgery (see Figure 1). Statistical significance was based on *p* < 0.05.

## 3. Results

A total of 26 cases met the inclusion criteria for this study. The median age at presentation was 11.2 years (range 2.9–17.1), and the median body weight was 4.4 kg (range 2.5–7.6). Signalment and presenting clinical signs are summarized in Table 1.

### 3.1. Pre-Operative Clinical Findings

The most common presenting signs were anorexia and vomiting (24/26 cats), followed by icterus and weight loss (13/26 cats). Eighteen cats presented more than seven days after the onset of clinical signs, and eight cats within seven days of the onset of clinical signs. Ten cats were reported to have one or more previously diagnosed ongoing comorbidities. These included cardiac disease (*n* = 4, hypertrophic cardiomyopathy [HCM] identified on cardiac ultrasound), renal insufficiency (*n* = 4), hepatic disease (cholangiohepatitis without biliary obstruction, based on blood work and abdominal ultrasound examination) recognized previously (*n* = 3), lower urinary tract infection (*n* = 2), diabetes mellitus (*n* = 1), otitis externa and mild bronchitis (*n* = 1). Other findings included nephroliths (*n* = 1), and bilateral chronic nephropathy with infarcts (*n* = 1).

### 3.2. Pre-Operative Medical Treatments

All cats had received medical treatment specific to EHBO for a median period of 26 days, (range, 4–394) prior to surgery, including broad-spectrum antimicrobials (*n* = 24), intravenous fluid therapy (*n* = 19), antiemetic medications (*n* = 18) and analgesic medications (*n* = 15). Other treatments included ursodeoxycholic acid (UDCA) (*n* = 11), S-Adenosylmethionine (SAMe) (*n* = 8) and parenteral vitamin K (*n* = 6).

### 3.3. Pre-Operative Blood Testing

Pre-operative blood testing was performed in all 26 cats. Not all cats had the same testing regime, which varied over the course of this study. Neutrophilia (14/23), leukocytosis (12/24), anemia (12/26) and monocytosis (9/23) were the most common abnormalities on hemograms. Elevations in gamma glutamyl transferase (GGT) (13/19), alanine amino transferase (ALT) (15/25) and total bilirubin (TBil) (14/24) were the most common abnormalities in serum biochemistry. Other abnormalities included elevations in alkaline phosphatase (ALP) (11/25), amylase (8/23) and cholesterol (8/24). A total of 3/26 cats had normal blood work despite bile duct obstructions later being identified via pre-operative ultrasound examination and intra-operative surgical findings.

Coagulation profiles were performed in 16/26 cats. Testing of both Prothrombin time (PT) and activated partial thromboplastin time (aPTT) was performed in 12 cats. Seven had prolonged aPTT and normal PT, while the other five had normal values for both parameters. Four other cats were tested for PT only, one of which had an elevated PT value.

### 3.4. Pre-Operative Imaging (Hepatobiliary)

Abdominal ultrasonography was performed in all 26 cats; 19 of these were performed by a board-certified internist or criticalist, and 7 by general practitioners. Abnormal findings included the following: distended gallbladder (*n* = 18), bi-lobed gallbladder (*n* = 2), thickened gallbladder wall (*n* = 13), sediment in the gallbladder (*n* = 15), and distended CBD (*n* = 8, diameter range 5 mm to 16.2 mm; normal < 5 mm [22,34]). Eight cats were reported to have cholelithiasis including cats with choleliths in both the gallbladder and CBD (*n* = 4), 3 cats presented with choledocholiths only and one cat with cholecystoliths only. Hepatic changes were reported in 11 cats, including heterogeneous parenchymal changes (*n* = 8), dilated hepatic ducts (*n* = 3), hepatomegaly (*n* = 2), mass-like lesion adjacent to the biliary tree (*n* = 1), cystic lesion within the liver (*n* = 1) and coarse liver architecture (*n* = 1). Pancreatic changes were documented in ten cats, with nine being consistent with pancreatitis and one reportedly presenting with a mass-like lesion within the pancreatic body. Free peritoneal effusion was noted in 11 cats, and among these, a ruptured gall bladder and bile peritonitis were surgically confirmed in one cat. In the other cases, the free fluid accumulation resulted from regional inflammation, based on cytologic examination. Bile leakage from the biliary tree into the peritoneal space was ruled out in these cases, as the sampled abdominal fluid contained TBil that did not exceed twice the serum levels of TBil.

### 3.5. Anesthesia Records

Complete anesthesia records were available for review in 18/26 cats, with eight incomplete anesthetic records. Peri-operative blood pressure was monitored primarily using an oscillometric method (Cardell^®^ Touch Veterinary Monitor, 8013-003; Midmark Co., Dayton, OH, USA; Manufacturer: Lutech Veterinary Industries Inc., Ronkonkoma, NY, USA). Fourteen of these eighteen cats had one or more episodes of hypotension intraoperatively. Hypotension management included the administration of up to four crystalloid fluid boluses of 5 mL of Lactated Ringers solution per Kg of body weight (*n* = 7), administration of a continuous rate infusion of dopamine (*n* = 6), administration of one or more boluses of colloids (6% hetastarch (Voluven^®^ 6% hydroxyethyl starch 130/0.4 in 0.9% sodium chloride injection) (*n* = 4), reduction in systemic anesthetic gas (Isoflurane in all cases) flow, and the use of alternative analgesic agents, such as administration of fentanyl and ketamine (*n* = 3). Other measures included administration of atropine (*n* = 3) and glycopyrrolate (*n* = 2). The measure of effective hypotension management was an improvement in MAP above 60 mm Hg.

The records of post-extubation blood pressure were available for review in 14 cats. Blood pressure was monitored primarily using an oscillometric device, and if found through repeat testing to be hypotensive, was reviewed with a Doppler (Doppler Medical Electronics 811-B; Parks^®^ Medical Electronics Inc., Aloha, OR, USA) device. Five of the fourteen cats were hypotensive post-operatively. These five cats deteriorated further, dying prior to discharge, with three cats experiencing cardiac arrest and two cats being euthanized following clinical deterioration.

### 3.6. Blood Product Administration

Fresh frozen plasma (FFP) was administered to four cats, including two intra-operative and two post-operative transfusions due to hypotension (MAP < 60 mmHg). FFP was given primarily as a management tool for hypotension in these cases, though possible benefits in terms of coagulopathy status and hypoproteinemia were also noted. Two cats received both FFP and pRBCs. Given the inherent risk of feline blood transfusion whereby type B cats receiving type A blood will often have fatal reactions, all blood transfusions were feline-specific, typed, and cross-matched. Corticosteroid administration is not part of this hospital’s feline blood transfusion protocol, and no reactions attributable to blood product administration were noted. Blood transfusions, including packed red blood cells (pRBCs) (*n* = 9) and whole blood (*n* = 1), were given either prior to surgery (*n* = 2), intraoperatively (*n* = 2), or post-operatively (*n* = 6), for cats which had PCV < 16%, and were showing clinical signs related to the anemia such as extreme lethargy or increased resting respiratory rates.

### 3.7. Intraoperative Findings and Surgical Management

All surgical procedures were performed by one of three board-certified surgery specialists. A combination of pre- and intra-operative assessments determined the direct involvement of the gall bladder in the development of the biliary obstruction in 22 cats, prompting a decision to perform a cholecystectomy as part of their treatment. Six cats which were intra-operatively assessed to have CBD stenosis secondary to cholelithiasis, regional inflammation or scarring had choledochal stents placed. One cat had CBD stenting without cholecystectomy, and five cats had both cholecystectomy and choledochal stenting. A duodenotomy was performed in twenty-two cats, enabling retrograde flushing of the CBD via the major duodenal papilla (MDP) to ensure CBD patency prior to cholecystectomy. Choledochal stenting, if performed, was temporary using a 3.5Fr- 5Fr red rubber cannula placed retrograde from the major duodenal papilla, beyond the intramural portion and into the CBD, then sutured with absorbable monofilament to the duodenal mucosa [3]. In three cats, advanced neoplasia of the biliary system was suspected based on unambiguous intraoperative findings; so, no further surgery or histological assessment was pursued at the caregiver’s direction.

There was one case of intestinal foreign body obstruction at the level of MDP (1/21).

### 3.8. Histopathology Sampling

Tissue samples were collected at surgery from 23/26 cats, and 21/26 cats had histologic studies pursued. A further 2/26 cats which died in the immediate post-operative period also had histologic studies declined by their caregivers. Definitive diagnoses were thus not made in 5/26 cats. Twenty gall bladders were submitted for histopathology, confirming cholecystitis in all cases. Cholelithiasis was confirmed in 8/21 cats which had tissues examined. Gall bladder rupture was suspected at surgery and confirmed through histologic examination in one cat. Cholangiohepatitis/cholangitis and hepatitis were the major findings in 15/21 liver samples submitted for histopathology. Infiltration by inflammatory cells (mixed neutrophilic and lymphoplasmacytic infiltrate) in the portal and periportal regions causing bile duct dilatation and periductular fibrosis was also noted in chronic cases. Hepatic lipidosis was reported in two cats. Hepatobiliary neoplasia was confirmed histologically in three cats, with diagnoses of metastatic adenocarcinoma, cholangiocellular carcinoma and lymphoma. These cases had not had cytologic sampling prior to open surgical investigation.

### 3.9. Microbiological Testing

Intraoperative samples from 22 cats, including bile (*n* = 20), liver tissue (*n* = 5) and choleliths (*n* = 2), were collected and submitted for bacterial culture and sensitivity testing, i.e., aerobic and anaerobic culture (*n* = 16), anaerobic only (*n* = 4) and aerobic only (*n* = 2). Bile was not able to be collected in one cat as the gallbladder was completely empty because of compression from a mass-like lesion in the quadrate lobe. In total, 6 of 22 cats had a positive culture, which included *Escherichia coli* from three bile samples, *Enterococcus cecorum* from one bile sample, *Delftia acidovorans* from one bile sample and *Staphylococcus epidermidis* from one bile and liver tissue samples. None of the choleliths demonstrated a positive culture.

### 3.10. Post-Operative Management

A total of 5 of 26 cats died within one day of surgery. Of the remaining 21 cats, 18 became hyporexic or anorexic post-operatively within five days of the surgery. Three cats ate normally post-operatively. Other clinical signs were observed during the post-operative period, including vomiting (*n* = 3), diarrhea (*n* = 3), and icterus (*n* = 1).

Twenty cats had a total of 22 feeding tubes placed peri-operatively. All cats had their fluid and electrolyte abnormalities corrected prior to initiation of gradually incremental tube feeding. Four esophageal feeding tubes (EFT) were placed pre-operatively, and seven immediately post-celiotomy. Seven nasogastric tubes (NGT) were placed peri-operatively. Two cats with NGT placed pre-operatively had an EFT placed in exchange for the NGT peri-operatively. Complications related to the feeding tubes were reported (*n* = 3), including premature NGT removal (*n* = 1), premature EFT removal (*n* = 1) and mild nasal discharge following NGT removal (*n* = 1), which resolved within one week without treatment.

Post-operative blood testing was not universally performed, and the decision to repeat blood testing was dependent on how cats were progressing clinically. Non-regenerative anemia (18/21) and neutrophilic leukocytosis (13/21) were the most common findings on a hemogram in cats surviving more than 24 h post-operatively. ALT elevation (16/21) was also common in cats tested in the post-operative period.

### 3.11. Outcomes

The etiology of EHBO was determined in most cats (21/26) by a combination of surgical and histopathological findings. The underlying disorders fell mainly into two main categories, inflammatory complexes (14/21) and neoplastic obstruction (6/21), with a single case of a duodenal foreign body obstruction (1/21).

The MST for cats was 86 days (range, 0–1497) post-surgery (see Figure 1). An MST of 17 days (range, 2–520) in cats with malignancies was found compared to an MST of 1165 days (range, 61–2268) in EHBO resulting from inflammatory complexes (see Table 2 for a summary of these findings).

In total, 18 of 26 cats survived until discharge, with a median hospital stay of 6 days (range 1–11 days, mean. 6.5 days). Of the eight cats that died prior to discharge, five did so within 24 h of surgery (cardiac arrest *n* = 3, and euthanasia under general anesthesia (*n* = 2). Three cats died of peri-operative complications, including suspected septic shock (*n* = 1), thromboembolism (*n* = 1) and refeeding syndrome (*n* = 1) based on clinical and biochemistry findings. A total of 17 of the 26 cats survived beyond two weeks post-operatively. One cat, following a cholecystectomy, duodenotomy and retrograde CBD flush, was euthanized eight days post-operatively due to bile peritonitis (TBil of abdominal fluid > twice serum TBil) and systemic inflammatory response syndrome (SIRS). Four other cats died between two weeks and six months post-operatively. Of these, three cats were euthanized for neoplasia of the biliary tree (*n* = 2) and severe cholangiohepatitis (*n* = 1) that failed to respond to medical treatment. One cat died from heart failure.

Half of the cats (13/26) survived more than six months after surgery. Of these 13 cases, complete resolution of clinical signs was reported in 8/13 cats. The other 5/13 showed intermittent vomiting, inappetence and/or diarrhea, with four of these five cats receiving intermittent medical treatments, including antimicrobial and antiemetic drugs. One cat had a second biliary surgery due to relapsing cholangiohepatitis secondary to an apparent stricture of the terminal CBD, with placement of a permanent self-expanding metallic CBD stent via duodenotomy 602 days following the first surgery. This cat remained clinically normal up until the time of writing two years later.

### 3.12. Variables Associated with Outcomes

Of the 14 cats with pre-operative hyperbilirubinemia, nine cats died in the immediate post-operative period, with only three cats surviving for longer than six months. There was a statistically significant association between pre-operative hyperbilirubinemia and poor short-term (*p* = 0.002) and long-term survival (*p* = 0.036), where pre-operative hyperbilirubinemia is associated with a decrease in the odds of surviving in the short term (odds ratio [OR] = 0.03, 95% CI (0.001, 0.57)) and in the long term (OR = 0.10, 95% CI (0.01, 0.69)).

Post-operative (following extubation) hypotension was also found to be statistically associated with poor short-term (*p* = 0.003) and long-term survival (*p* = 0.021). Post-operative hypotension was associated with a decrease in the odds of surviving in the short term (OR = 0.02, 95% CI (0.001, 0.47)) and in the long term (OR = 0.03, 95% CI (0.001, 0.77)).

There was a statistical association between malignancy and poor long-term survival (*p* = 0.047). Cats with a malignancy had decreased odds of surviving in the long term (OR = 0.08, 95% CI (0.01, 0.98)).

See Table 3 and Table 4.

## 4. Discussion

This study identified potential prognostic factors which may affect the survival of cats undergoing surgery for EHBO. Pre-operative hyperbilirubinemia and immediate post-operative hypotension were significantly associated with reduced short-term and long-term survival. This study also found that cats with neoplasia of the biliary tract carry a poor prognosis for survival when undergoing surgery for biliary obstruction compared with those with inflammatory disease as the cause for their biliary obstruction. The association between malignancy of the hepatobiliary system and poor long-term outcome corroborates previous studies from Mayhew et al. in 2002, Buote et al. in 2006 and Simpson et al. in 2021, which demonstrated poor prognosis in cats with biliary obstructions associated with malignancies [6,7,12].

The collective term ‘Inflammatory complexes’ describes a wide range of inflammatory conditions, including cholangitis/cholangiohepatitis, pancreatitis and enteritis, or triaditis, when inflammation is noted in all three regions simultaneously [1,35,36]. Sharing such similar pathogeneses, these entities were grouped for this study. Cholangitis and cholangiohepatitis reflect the progressive stages of one disease [35]. Although the etiopathogenesis of these three disorders remains unclear, speculation of an immune-mediated mechanism and enteric bacterial reflux into the shared opening of the pancreatic and CBD have both been suggested [35,36].

Cholelithiasis or choledocholithiasis are considered two of the primary etiologies of EHBO in some studies [8,10,12,22,37], although they may be a consequence and a component of an inflammatory process [1]. The tendency for cholelithogenicity in cats remains unclear. It is evident that the formation of bile sludge, bile sediment and choleliths is often present simultaneously with inflammation, which modifies bile composition and can alter bile flow. Increases in ductular secretion of bicarbonate and mucin result in thickened and inspissated bile secretions [23].

Multiple other clinical factors were examined which did not affect the survival of cats undergoing surgery for EHBO.

Anorexia and vomiting were reported to be the most common clinical signs in cats with EHBO, which were seen in more than 90% of cats but are not specific to EHBO. Icterus, though more specific to EHBO, was only seen in half of the cats.

Elevated serum hepatic and cholestatic enzyme activities, including GGT, ALT, ALP, TBil, were common findings amongst cats in the study. The association between pre-operative hyperbilirubinemia and poor short-term and long-term survival is noteworthy. Bacterial endotoxemia resulting from poor flow of bile into the gastrointestinal tract is thought to be an important mediator of mortality in patients with complete biliary obstruction, as well as the direct renal cytotoxicity of bilirubin, which may in part explain these findings. This correlates with similar studies in dogs and people undergoing hepatobiliary surgeries [38,39,40,41,42,43]. Three cats had normal blood work despite findings of biliary obstruction at surgery, indicating the importance of investigating feline patients presenting with signs which might be related to the hepatobiliary system with blood work and abdominal imaging.

Non-regenerative anemia and anorexia were two of the most common post-operative clinical findings, with most of the patients (21/23) surviving more than 24 h post-operatively presenting with these signs. Post-operative anemia could be attributed to intra-operative blood loss, coagulopathy, and/or hemodilution with peri-operative fluid therapy. In this study, nearly half of the cats required blood transfusion. Given these findings, it would appear prudent to ensure that there is the availability of blood products, blood typing and cross-matching for cats undergoing surgery for EHBO.

Nearly three-quarters of cats required enteral feeding tube placement to allow for adequate caloric intake peri-operatively. Recommendations for a feeding tube to be placed peri-operatively have also been made in other publications [1,7].

A large proportion (13/19) of cats with anesthesia records available for review were hypotensive peri-operatively. This may have resulted from numerous factors, including inadequate intravenous fluid resuscitation, inhalational anesthetic administration, prolonged anesthesia, vagal responses, renal toxicity secondary to elevated serum bilirubin levels or development of SIRS and/or sepsis [41,44]. Given the retrospective nature of our study, it was not always possible to determine the cause(s) of hypotension in individual cases. However, it is noteworthy that all cats with immediate post-operative hypotension failed to survive to discharge. The finding that post-operative hypotension led to reduced short-term and long-term survival in this study is similar to studies involving dogs, in which post-operative hypotension following cholecystectomy was found to be significantly associated with poor outcome, with a 20-time higher risk of death [45]. There is research to support significant variability between Doppler and oscillometric blood pressure monitoring in conscious cats [46]. Hypotension was confirmed by repeat testing and by further confirmation using a Doppler device.

This study had several limitations, including its retrospective and descriptive study design, as well as the small sample size. Moreover, case management and surgical technique varied between surgeons as well as over time. Feline EHBO is rare, representing only 0.09% of all feline patients presented to this hospital over the same period. Low case numbers are one of the main factors precluding the identification of specific risk factors for peri-operative complications and this brings into question whether the factors identified herein are truly relevant. Histological investigations were not carried out in five of the twenty-six cases, further impacting the already small sample. Likewise, without necropsy, pathologies restricted to non-resectable regions were not likely to be detected.

This study has clinical relevance since it has identified potential clinical factors, which when identified may worsen the prognosis for cats undergoing surgery for EHBO. These findings were apparent despite the potential for the small sample size to result in low power. This study has similar case numbers to those in the existing body of literature, and clearly, the statistical tests had sufficient power to detect the reported associations. Nonetheless, the small sample does limit the generalizability of the results of this study. This study was always intended as a starting point for further studies in the area involving larger cohorts.

## 5. Conclusions and Clinical Relevance

Pre-operative hyperbilirubinemia and immediate post-operative hypotension were identified as possible poor prognostic factors in cats undergoing surgery for EHBO, being associated with reduced short-term and long-term survival. Cats with EHBO caused by malignancies have poor rates of survival from surgery compared with those with inflammatory-complex-related obstructions. The prognosis for cats undergoing extra-hepatic biliary surgeries should be considered guarded until malignancy can be ruled out.

## Figures and Tables

**Table 1:** Signalments and presenting signs.

Variable	Cats (n)	Cats (%) ^a^
Sex		
Male Neutered	13	50
Female Spayed	13	50
Body Condition Score		
1 to <4 (underweight)	7	27
4–5 (ideal)	10	38
>5 to 9 (overweight)	9	35
Breed		
Domestic Short Hair	12	46
British Short Hair	5	19
Exotic	5	19
Persian	2	8
Scottish Fold	1	4
Norway Forest	1	4
Duration of clinical signs prior to surgery		
<7 days	7	27
8–30 days	5	19
>30 days	13	50
Unknown	1	4
Presenting clinical signs		
Vomiting	24	92
Anorexia	24	92
Icterus	13	50
Weight loss	13	50
Lethargy	13	50
Dehydration	13	50
Fever ^b^	10	38
Abdominal pain ^c^	9	35
Diarrhea	8	31
Distended abdomen	5	19
Pale mucus membrane	5	19
Polyuria, Polydipsia	4	15
Dyspnea	2	8
Constipation	2	8

^a^ Total number of cats is 26. ^b^ Rectal temperature > 39.2 °C [102.5 °F]. ^c^ Abdominal pain (yes/no) was not recorded for one cat.

**Table 2:** Etiology, histopathological findings, presence of obstructive cholelithiasis, surgical procedure(s) performed, survival time and cause of death in each case.

Case No.	Etiology	Histological Findings	Presence of Cholelithiasis	Surgical Procedure(s)	Survival Time (Days)	Cause of Death ^f^
1	ID	CHP, cholecystitis, enteritis	Yes	C, D	1497	Unknown
2	ID	Cholangitis	Yes	C, D	1165	Unknown
3	ID	CHP, cholecystitis	Yes	C, D	904 ^g^	n/a
4	ID	Cholecystitis, cholangitis	Yes	C, D	439 ^g^	n/a
5	ID	Cholecystitis, pancreatitis	Yes	C, D	414	Arrested—DKA
6	ID	Cholecystitis, cholangitis	No	C, D	275 ^g^	n/a
7	ID	CHP, cholecystitis	No	C, D	141 ^g^	n/a
8	ID	Hepatic congestion, cholecystitis	No	C, D	104 ^g^	n/a
9	ID	CHP, cholecystitis, hepatic lipidosis	No	C, D	8	Euth (Bile +/− septic peritonitis)
10	ID	Cholecystitis, triaditis	No	C	86	Arrested—HCM
11	ID	Cholecystitis, hepatitis	No	C, S, D	521 ^g^	n/a
12	ID	CHP, cholecystitis	No	C, S, D	50 ^g^	n/a
13	ID	CHP, cholecystitis, hepatic lipidosis	No	C, S, D	6	Euth (Refeeding Syndrome)
14	ID	Cholecystitis, hepatitis, enteritis	No	C, S, D	1	Arrested (POC)
15	Benign N	Cystadenoma	No	C, D	1435	Euth (Chronic CHP)
16	Malignant N	T-cell lymphoma	Yes	C, D	40	Euth (Severe CHP & NRC)
17	Malignant N	T-cell lymphoma	No	C, D	520	Euth (NRC)
18	Malignant N	Adenocarcinoma (gallbladder)	No	S, D	17	Arrested (NRC)
19	Malignant N	Cholangiocellular carcinoma	No	C, D	2	Euth (POC)
20	Malignant N	Metastatic adenocarcinoma (pancreas, gastrointestinal tract)	No	C, S, D	4	Euth (POC)
21	Duodenal FB	CHP, cholecystitis, enteritis	No	C, D	227 ^g^	n/a
22	Indeterminate	Unknown	Yes	C, D	0	Arrested (POC)
23	Indeterminate	Unknown	No	C, D	0	Arrested (POC)
24	Indeterminate	Unknown	Unknown	Not performed	23	Euth (AND)
25	Indeterminate	Unknown	Unknown	Not performed	0	Euth (AND)
26	Indeterminate	Unknown	Unknown	Not performed	0	Euth (AND)

AND: Advanced neoplastic disease; C: Cholecystectomy; CHP: Cholangiohepatitis; D: Duodenotomy; DKA: Diabetic ketoacidosis; Euth: Euthanized; HCM: Hypertrophic cardiomyopathy; ID: Inflammatory disease; S: Stenting of the common bile duct; N: Neoplasia; NRC: Neoplasia-related complications; n/a: Not applicable; POC: Post-op complications; ^f^ Possible cause of death is based on medical records. Note that autopsy was not performed in this study and the true cause of death was not confirmed; ^g^ Still alive at time of writing.

**Table 3:** Associations between prognostic factors and short-term ([3] 2-week, <6-month) survival.

Characteristic	n	Short-Term Survival		*p*-Value	OR	95% CI
		Yes	No			
Anemia pre-op n (%)						
Yes	12 (46.2)	6 (23.1)	6 (23.1)	0.218	0.27	0.05–1.50
No	14 (53.8)	11 (42.3)	3 (11.5)			
Neutrophilia pre-op n (%)						
Yes	14 (60.9)	9 (39.1)	5 (21.7)	1.000	1.44	0.26–7.96
No	9 (39.1)	5 (21.7)	4 (17.4)			
Monocytosis pre-op n (%)						
Yes	9 (39.1)	4 (17.4)	5 (21.7)	0.383	0.32	0.06–1.85
No	14 (60.9)	10 (43.5)	4 (17.4)			
Elevated ALT pre-op n (%)						
Yes	15 (60.0)	9 (36.0)	6 (24.0)	0.691	0.64	0.12–3.53
No	10 (40.0)	7 (28.0)	3 (12.0)			
Elevated Total Bilirubin pre-op n (%)						
Yes	14 (58.3)	5 (20.8)	9 (37.5)	0.002	0.03	0.001–0.57
No	10 (41.7)	10 (41.7)	0 (0.0)			
Clotting profile ^h^ n (%)						
Normal	8 (50.0)	7 (43.8)	1 (6.3)	0.119	11.67	0.92–147.57
Abnormal	8 (50.0)	3 (18.8)	5 (31.3)			
Leukocytosis post-op n (%)						
Yes	13 (68.4)	9 (47.4)	4 (21.1)	0.255	0.16	0.01–3.56
No	6 (31.6)	6 (31.6)	0 (0.0)			
Neutrophilia post-op n (%)						
Yes	11 (57.9)	9 (47.4)	2 (10.5)	1.000	1.50	0.16–13.75
No	8 (42.1)	6 (31.6)	2 (10.5)			
Hyperglycemia post-op n (%)						
Yes	7 (36.8)	6 (31.6)	1 (5.3)	1.000	2.00	0.17–24.07
No	12 (63.2)	9 (47.4)	3 (15.8)			
Elevated ALP post-op n (%)						
Yes	8 (42.1)	5 (26.3)	3 (15.8)	0.262	0.17	0.01–2.04
No	11 (57.9)	10 (52.6)	1 (5.3)			
Elevated Total Bilirubin post-op n (%)						
Yes	11 (57.9)	8 (42.1)	3 (15.8)	0.603	0.38	0.03–4.55
No	8 (42.1)	7 (36.8)	1 (5.3)			
Hypalbuminemia post-op n (%)						
Yes	7 (35.0)	5 (25.0)	2 (10.0)	0.587	0.45	0.05–4.21
No	13 (65.0)	11 (55.0)	2 (10.0)			
Hypotension Intra-op n (%)						
Yes	14 (77.8)	8 (44.4)	6 (33.3)	1.000	0.44	0.04–5.41
No	4 (22.2)	3 (16.7)	1 (5.6)			
Hypotension Post-op n (%)						
Yes	5 (35.7)	0 (0.0)	5 (35.7)	0.003	0.02	0.001–0.47
No	9 (64.3)	8 (57.1)	1 (7.1)			
Malignancy n (%)						
Yes	5 (23.8)	3 (14.3)	2 (9.5)	0.553	0.35	0.04–3.08
No	16 (76.2)	13 (61.9)	3 (14.3)			
Inflammation n (%)						
Yes	14 (66.7)	11 (52.4)	3 (14.3)	1.000	1.47	0.18–11.72
No	7 (33.3)	5 (23.8)	2 (9.5)			

^h^ Some cats were tested for PT but not aPTT. Cats with abnormal results in either PT or aPTT would be considered abnormal. Abbreviations: ALP—alkaline phosphatase, aPTT—activated partial thromboplastin time, CI—confidence interval, OR—odds ratio, PT—prothrombin time.

**Table 4:** Associations between prognostic factors and long-term ([3] 6-month) survival ^i^.

Characteristic	n	Long-Term Survival		*p*-Value	OR	95% CI
		Yes	No			
Anemia pre-op n (%)						
Yes	12 (46.2)	4 (15.4)	8 (30.8)	0.238	0.28	0.05–1.41
No	14 (53.8)	9 (34.6)	5 (19.2)			
Neutrophilia pre-op n (%)						
Yes	14 (60.9)	7 (30.4)	7 (30.4)	1.000	1.25	0.23–6.71
No	9 (39.1)	4 (17.4)	5 (21.7)			
Monocytosis pre-op n (%)						
Yes	9 (39.1)	2 (8.7)	7 (30.4)	0.089	0.16	0.02–1.08
No	14 (60.9)	9 (39.1)	5 (21.7)			
Elevated ALT pre-op n (%)						
Yes	15 (60.0)	8 (32.0)	7 (28.0)	1.000	1.14	0.23–5.67
No	10 (40.0)	5 (20.0)	5 (20.0)			
Elevated Total Bilirubin Pre-op n (%)						
Yes	14 (58.3)	4 (16.7)	10 (41.7)	0.036 ^i^	0.10	0.01–0.69
No	10 (41.7)	8 (33.3)	2 (8.3)			
Clotting profile ^j^ n (%)						
Normal	8 (50.0)	5 (31.3)	3 (18.8)	0.619	2.78	0.37–21.03
Abnormal	8 (50.0)	3 (18.8)	5 (31.3)			
Leukocytosis post-op n (%)						
Yes	13 (68.4)	7 (36.8)	6 (31.6)	0.333	0.23	0.02–2.59
No	6 (31.6)	5 (26.3)	1 (5.3)			
Neutrophilia post-op n (%)						
Yes	11 (57.9)	7 (36.8)	4 (21.1)	1.000	1.05	0.16–6.92
No	8 (42.1)	5 (26.3)	3 (15.8)			
Hyperglycemia post-op n (%)						
Yes	7 (36.8)	6 (31.6)	1 (5.3)	0.333	4.29	0.39–47.63
No	12 (63.2)	7 (36.8)	5 (26.3)			
Elevated ALP post-op (%)						
Yes	8 (42.1)	4 (21.1)	4 (21.1)	0.319	0.22	0.03–1.75
No	11 (57.9)	9 (47.4)	2 (10.5)			
Elevated Total Bilirubin post-op n (%)						
Yes	11 (57.9)	6 (31.6)	5 (26.3)	0.177	0.17	0.02–1.91
No	8 (42.1)	7 (36.8)	1 (5.3)			
Hypalbuminemia post-op n (%)						
Yes	7 (35.0)	3 (15.0)	4 (20.0)	0.174	0.23	0.03–1.62
No	13 (65.0)	10 (50.0)	3 (15.0)			
Hypotension Intra-op n (%)						
Yes	14 (77.8)	6 (33.3)	8 (44.4)	0.576	0.25	0.02–3.04
No	4 (22.2)	3 (16.7)	1 (5.6)			
Hypotension Post-op n (%)						
Yes	5 (35.7)	0 (0.0)	5 (35.7)	0.021	0.03	0.001–0.77
No	9 (64.3)	7 (50.0)	2 (14.3)			
Malignancy n (%)						
Yes	5 (23.8)	1 (4.8)	4 (19.0)	0.047	0.08	0.01–0.98
No	16 (76.2)	12 (57.1)	4 (19.0)			
Inflammation n (%)						
Yes	14 (66.7)	10 (47.6)	4 (19.0)	0.346	3.33	0.50–22.14
No	7 (33.3)	3 (14.3)	4 (19.0)			

^i^ One cat was only 70 days post-op. Its status for six-month survival was unknown and therefore excluded. ^j^ Some cats were tested for PT but not aPTT. Cats with abnormal results in either PT or aPTT would be considered abnormal. Abbreviations: ALP—alkaline phosphatase, aPTT—activated partial thromboplastin time, CI—confidence interval, OR—odds ratio, PT—prothrombin time.

## Data Availability

Any information not included herein can be made available by contacting the corresponding authors.
